# Case report: Lingering post-concussive symptoms in a pediatric patient with presumed Ehlers-Danlos syndrome

**DOI:** 10.3389/fped.2022.937223

**Published:** 2022-11-04

**Authors:** Tala Maris Curry, Mitra Esfandiarei, Theresa Currier Thomas, Reena Gogia Rastogi

**Affiliations:** ^1^ Department of Child Health, College of Medicine-Phoenix, University of Arizona, Phoenix, AZ, United States; ^2^ Neurotrauma and Neurochemistry Research Laboratory, Barrow Neurological Institute, Phoenix Children's Hospital, Phoenix, AZ, United States; ^3^ Department of Biomedical Sciences, College of Graduate Studies, Midwestern University, Glendale, AZ, United States; ^4^ Department of Basic Medical Sciences, College of Medicine-Phoenix, University of Arizona, Phoenix, AZ, United States; ^5^ Pediatric Headache Program, Barrow Neurological Institute, Phoenix Children's Hospital, Phoenix, AZ, United States

**Keywords:** concussion, pediatric, Ehlers-Danlos syndrome, case report, post-concussive symptoms

## Abstract

**Background:**

Connective tissue disorders such as Ehlers-Danlos Syndrome (EDS) can affect collagen and elastin content and structure, including weakening of tissues and vasculature, thus contributing to multiple systemic manifestations. Prior research has successfully focused on peripheral life-threatening manifestations resulting in increased life expectancy, yet clinical observations have warranted investigation of neurological vulnerability, where little is known. Compromised brain tissues and cerebrovasculature could leave these patients vulnerable to mild traumatic brain injury (TBI), with increased severity and duration of post-concussive symptoms and delayed recovery. Clinical reports in adults indicate that higher severity of symptoms after a mild TBI, such as a concussion, can unmask connective tissues disorders leading toward diagnosis. This clinical case report is an example of a pediatric patient with presumed Ehlers-Danlos syndrome who demonstrates increased vulnerability to mild TBI/concussion.

**Patient:**

A pediatric female patient presents with unexplained lingering post-concussive symptoms, including trouble sleeping, nausea, frontal headaches, dizziness, visual changes, fatigue, and left-sided weakness more than 6 months post-mild concussion. Patient history of hypermobility, joint derangement, soft tissue mobility, and bruising suggests a potential diagnosis of Ehlers-Danlos syndrome, which may explain symptom severity and length of recovery.

**Discussion:**

This case is the first documented instance of increased vulnerability to TBI in a pediatric patient with presumed Ehlers-Danlos syndrome. It highlights the need for awareness and prevention of injury in this vulnerable patient population, suggests more targeted therapeutic intervention for recovery, and demonstrates the need for preclinical research evaluating the influence of genetic mutations associated with connective tissue disorders on the central nervous system.

## Introduction

Connective tissue disorders are systemic diseases with pathological effects on tissues and organs particularly high in collagen and elastin content, such as Ehlers-Danlos syndrome (EDS), Marfan syndrome (MFS), and Loey's-Dietz syndrome (LDS). EDSs comprise a group of autosomal-dominant systemic connective tissue disorders. At least thirteen phenotypic types of EDSs have been identified with a combined prevalence of 1 in 2500–5000 (1–3). Overall, EDS has common manifestations of skin hyperextensibility, joint hypermobility, vascular abnormalities, and fragile tissues. In addition, patients may experience neurological and spinal manifestations such as headaches, migraines, and craniocervical instability (4–6). Due to the broad phenotypic spectrum, less severe forms of EDS are likely to go undiagnosed and may only be diagnosed after an unmasking event. An unmasking event is when a life-altering physiological stressor induces increased symptomology, indicating an underlying disorder. Of the thirteen types of EDS, hypermobile EDS is the most common form and has an unknown genetic basis (1, 3, 7). Conversely, other types of EDS result from genetic defects such as haploinsufficiency of the tenascin-XB (*TNXB*) gene, mutations in *COL5A1* (chromosome 9q34) and *COL5A2* (chromosome 2q31) genes, and more genetic mutations that primarily effect extracellular matrix proteins (8, 9). Thus diagnosis of EDS can be difficult and genetic testing is recommended. In order to meet hypermobile EDS criteria, the patient must display generalized joint hypermobility, two or more criteria including connective tissue manifestations, family history, or musculoskeletal complications, and exclusion of other connective tissue disorders (8). These common manifestations seen in EDS, specifically fragile tissues and weakened vasculature, have not been evaluated in the context of TBI, where this patient population is more vulnerable to lasting post-concussive symptoms after a mild injury.

TBI disrupts brain function by imparting acceleration-deceleration/rotation forces to the brain, causing mechanical injury to the neurons, vasculature, and glia, instigating an extensive range of acute, subacute, and persisting post-concussive symptoms (PCS). Over 69 million TBIs are reported annually worldwide, making it the most common acquired brain injury (10, 11). Furthermore, nearly 80% are mild TBIs, which are vastly under-reported. Experts predict the actual incidence could be as high as ∼50% of the world's population acquiring a head injury in their lifetime (12). Though a broad age range is affected, TBI is the leading cause of death and disability in pediatric patients (10, 13). In addition, menstrual cycling females are at higher risk for increased number, severity, and duration of symptoms secondary to TBI (14, 15). Mild TBI (mTBI) causes symptoms ranging from mild to severe, including headaches, mood changes, anxiety, fatigue, sleep disruption, memory impairment, difficulty learning, and more (12). A mTBI is diagnosed according to the Glasgow Coma Scale, measuring eye-opening, verbal response, and motor response to test consciousness, as a score of 13–15 (16). Symptom resolution in the pediatric patient population is around one week to one month, but can also persist longer, potentially causing long-term neurological impairment (17). The leading causes of mTBI are unintentional falls, sports-related injuries, motor vehicle accidents (including ATV, motorcycles, electric scooters, e-bikes, etc.), assault, and blast exposures, as well as the possibility of being unintentionally struck by or against an object. TBI can result in acute and persisting cognitive, emotional, sensory deficits, sleep disorders, and behavioral abnormalities, with 43% reported TBI survivors developing long-term effects (11, 17–19). Interestingly, mTBI has been reported to unmask EDS in 7 adult EDS patients, who experienced a slower and less complete recovery from the injury (3). This case report supports a similar incident in the pediatric population, indicating increased vulnerability of connective tissue disorder patients to acquired neurological injury, resulting in persisting post-concussive symptoms that diminish the patient's quality of life.

## Patient case

**Figure 1:**
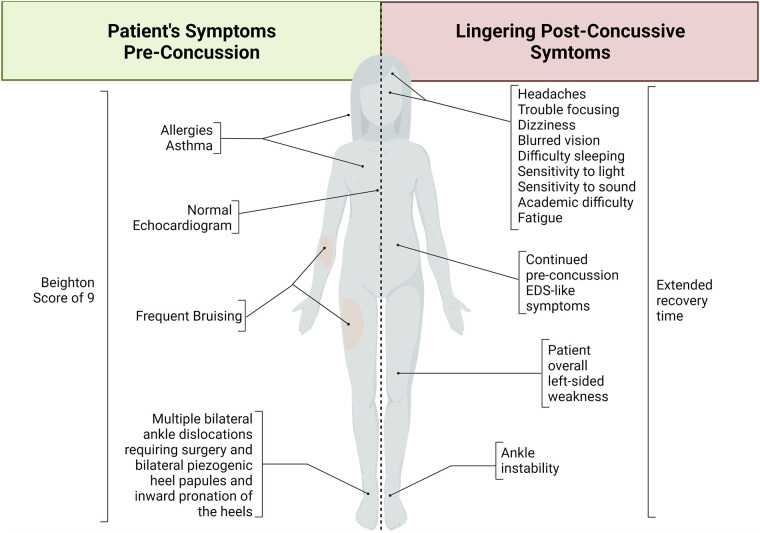
Schematic summary of Ehlers-Danlos syndrome-like symptoms before concussion and lingering post-concussive symptoms. This patient demonstrated various Ehlers-Danlos syndrome-like symptoms before her concussive event. After concussion, the patient developed various symptoms that continued long after the expected recovery time. Taken together, this patient case suggests a necessary diagnosis, support, and care of EDS as well as indicating increased vulnerability to increased symptom severity and longevity after traumatic brain injury in this patient population.

The presenting patient is a 15-year-old assigned female at birth who suffered a concussion without loss of consciousness secondary to an accidental kick to the face. Before concussion, the patient experienced multiple dislocations and displayed symptoms of hyperextensibility and hypermobility. Due to the severity and frequency of dislocations, she required ankle surgery bilaterally and was counseled to seek an assessment for EDS. EDS had not been confirmed at the time of concussion, but the patient received an echocardiogram to begin the assessment for EDS, which was normal. Due to the patient's age and unknown form of EDS, a normal echocardiogram does not exclude the patient from an EDS diagnosis. The patient does not have a family history of EDS or any other connective tissue disorders nor a history of concussion. Lack of family history of EDS does not disqualify diagnosis. She presents with a history of asthma and a family history of allergies, heart attack, hypertension, asthma, diabetes mellitus, and frequent headaches. A hypermobility screening suggested that several of the patient's symptoms are consistent with EDS/hypermobility, including chronic pain (e.g., lower back, upper back, hips, shoulders, ankles, knees, and left wrist pain), dizziness sit-to-stand, abdominal pain, and frequent bruising. In addition, the patient displays bilateral piezogenic heel papules, bilateral inward-leaning pronation of the heels, and a 9 out of 9 on the Beighton hypermobility scale (Figure 1). This 9-point system is a common technique used to quantify joint laxity and hypermobility, where higher scores are indicative of hypermobility and associated syndromes such as EDS (20, 21).

**Figure 2:**
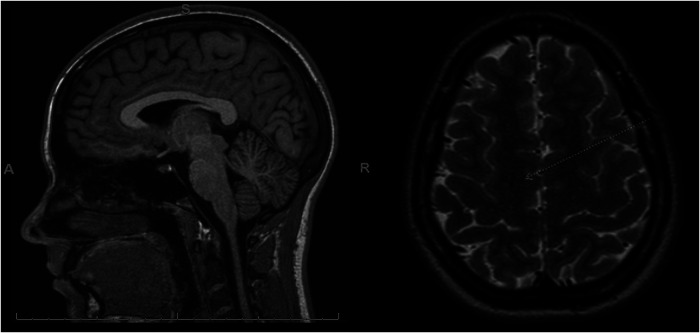
MRI post-concussive brain imaging of the patient. In this case, the patient received an MRI 2 weeks after injury. The MRI revealed an incidental right frontal developmental venous anomaly (white arrow) and was otherwise normal. No pre-concussive MRIs are available for comparison.

The patient is a competitive dancer and was accidentally kicked in the face during a routine, which resulted in a facial injury, bloody nose, dizziness, and headache. Immediately after the accident, the patient felt confused and disoriented with a headache that brought her to the emergency department. A maxillofacial CT revealed a left maxillary sinus fracture and minimally displaced nasal bone fracture, which did not require surgical intervention. One-week post-facial injury, the patient reported trouble sleeping, nausea, constant frontal headaches without relief from medications, dizziness sit-to-stand, dizziness while walking, trouble focusing, increased light sensitivity and blurred vision with difficulty tracking, and increased fatigue, thus requiring a neurology consult. During the consult, the patient displayed left-sided upper and lower extremity weakness and difficulty with balance testing, displaying a mild truncal sway and stepping off midline repeatedly with tandem gait forward, backward, and forward with eyes closed. A diagnosis of concussion was confirmed, and a brain MRI ordered, revealing no intracranial abnormality but showing an incidental right frontal developmental venous anomaly (Figure 2).

**Figure 3:**
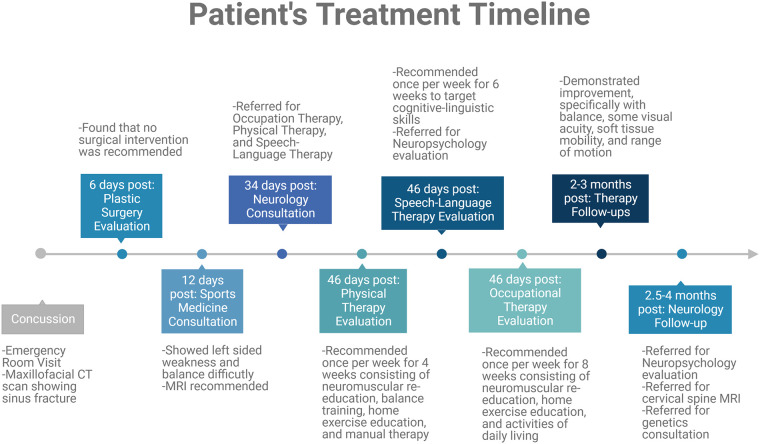
Timeline summary of therapeutic interventions relevant to this case report. This patient case required a therapeutic approach with various consultations and therapies recommended. These evaluations are summarized in this timeline.

A therapeutic approach was taken to help mitigate these lingering post-concussive symptoms (Figure 3). The following tests were performed during focused therapy to determine the patient's deficits. A lower extremity functional scale (LEFS) assessment was performed. This assessment is a patient-reported survey of everyday task abilities, where this patient had 95% function 3 months post-concussion. Further evaluation of the patient's left-sided weakness displayed impaired cervical and shoulder range of motion with decreased left forearm, shoulder, elbow, and wrist strength and coordination which improved with physical therapy but has not returned to pre-injury abilities. The post-concussion visual assessment demonstrated that the patient's visual perception was well below average for her age. Her oculomotor control, horizontal and vertical smooth pursuit, and horizontal and vertical saccades were all impaired for her age and compared to her pre-injury status. The patient's maximum difficulty was to converge with impaired coordination with the left side compared to the right, with a convergence of 22 cm.

The cognitive-linguistic evaluation led to premature fatigue during the assessment due to cognitive exertion. She also demonstrated slowed processing, word-finding difficulties, mood changes, and increased irritability. The patient's performance during the cognitive-linguistic exam also suggested difficulty with working memory within functional limits and moderate immediate and short-term memory deficits. She also demonstrated deficits in sustained attention, processing speed, response time, detailed recall, and displayed disorganized narratives. Approximately 4 months post-injury, the patient returned to dancing. She continued to experience left-sided weakness with ankle instability, dizziness, headaches, trouble focusing, and memory recall when loud noises, such as music playing. These factors affected her ability to achieve optimal performance and regain her quality of life.

At the time of this report, 6 months post-concussion, the patient still reports increased headaches, fatigue, left-sided weakness, dizziness, blurred vision, difficulty sleeping, increased sensitivity to loud noises, and trouble focusing with new-onset difficulty in academics (Figure 1). The patient and providers report some improvement through focused therapy, which was tolerated well by the patient. At this patient's age, the expected recovery time for a mild concussion is less than a month (17). Brain injury has been shown to unmask connective tissue disorders such as EDS (3). In addition, the left- sided weakness demonstrated is atypical of a mild concussion and may be attributable to EDS.

The patient's increased dysfunction and extended recovery time, in addition to joint hypermobility and musculoskeletal complications, warrant further evaluation of the patient for possible EDS by utilizing genetic testing, a more complete family history, and exclusion of alternative diagnoses (22). Though a follow-up of genetic testing and continued focused therapies were recommended, there were challenges associated with diagnosing and further treating this patient. This patient was referred to a geneticist to further this evaluation, but unfortunately has not been completed due to unknown circumstances where the patient's parent rescheduled the appointment on multiple different occasions and on the last attempt did not show up for the appointment. Thus, genetic testing is not available in this case. Furthermore, this patient was referred for neuropsychology evaluation, yet similarly did not show up for their scheduled appointment. Thus, the inability to follow-up with this patient prevents differing diagnoses or psychiatric evaluation.

## Discussion

EDS contributes to increased vulnerability to various injuries through increased bruising, bone fragility, and vascular abnormalities, with growing support for increased susceptibility to severe outcomes after an acquired neurological insult. It has been previously demonstrated that TBI in adult EDS patients results in longer recovery times, headaches, migraines, dizziness, changes in vision, fatigue, and more (3, 23). To the best of our knowledge, this patient is the first reported case of increased vulnerability to TBI in pediatric subclinical EDS. Previous reports of abnormally severe deficits from a mild TBI instigating and leading to an EDS diagnosis represent TBI's potential as an unmasking event for undiagnosed EDS (3, 23). This case represents a patient suspected to have EDS before injury, whose response to a mild TBI caused more abnormally severe and persistent symptoms, negatively impacting her quality of life. This case is another indicator that patients with EDS may be more susceptible to increased symptom severity secondary to TBI. In addition, life-threatening comorbidities associated with EDS such as aneurysms and blood vessel ruptures, often do not occur until adulthood, such that many patients go undiagnosed until these vascular insults occur. This case supports that increased vulnerability and life-altering symptoms can occur earlier in life before onset of these life- threatening comorbidities. In addition, this patient has returned to competitive dancing even though she is having lingering post-concussive symptoms, putting her at increased risk for second/repeated TBI that may worsen her symptom severity. Interestingly, visual complaints such as blurred vision lingering for months to years without notable physical changes to the visual system, such as symptoms displayed by this patient, correlates with previous reports of EDS and concussion co-diagnosis seen in adult patients (3). Considering this patient's increased risk for repetitive head injuries and thus traumatic encephalopathy syndrome (TES) leading to chronic traumatic encephalopathy (CTE), this case was evaluated using the National Institute of Neurological Disorders and Stroke diagnostic criteria for TES (24). The patient did not meet criteria for TES, as she does not have a history of multiple head injuries. This evaluation should be utilized in cases of repeated head injuries (24). An unmasking event may be potentially beneficial in diagnosing a connective tissue disorder patient and allowing them to seek treatment for their associated symptoms, thus increasing their life expectancy. However, it is noteworthy that lingering post-concussive symptoms alter the patient's quality of life and the length of recovery time is uncertain.

This patient case is remarkable due to the increased number and worsened symptoms displayed in a pediatric case. Difficulties in diagnosis and follow-up were faced in this patient case, yet this patient suggests that EDS and other connective tissue disorder patients, caregivers, and physicians should be aware of these more severe outcomes to raise awareness regarding prevention and more comprehensive treatments required for this patient population. This patient tolerated and progressed through multiple therapies, including occupational, physical, and speech-language therapy to target symptomatology seen in this case (Figure 3). It is recommended that a more comprehensive therapeutic approach that includes visual, physical, occupational, and cognitive therapies, among others, should be considered in this patient population to improve lingering post-concussive symptoms. For patients presenting with both abnormally severe and persistent symptoms after neurological insult and any other common symptoms associated with connective tissue disorders, a thorough review of the patient and family history and consultation with expert geneticists for an underlying diagnosis of EDS or other connective tissue disorders are highly recommended (3–5, 23, 25). Further, hypermobility (and other overt connective tissue disorder symptoms) may be a potential risk factor for/indicator of suboptimal recovery from TBI, and requires further investigation.

Preclinical and translational research is required to consider the mechanisms responsible for increased vulnerability to TBI in this connective tissue disorder patient population. Cerebrovascular and neurological investigations in preclinical connective tissue disorder models are minimal. The *Fbn1*^C1039G/+^ Marfan Syndrome (MFS) mouse model is one of the most well studied and characterized connective tissue disorder mouse models. One study has addressed the cerebrovasculature in this mouse model, where the middle cerebral artery (MCA) demonstrated increased wall/lumen hypertrophy and increased TGF-*β* and MMP-9 expression as well as elevated reactive oxygen species (ROS) production in the MCA (26). Another study reported that apolipoprotein E (ApoE)-deficient mice with the same Fbn1 mutation (Fbn1^C1039G/+^) (ApoE-/-Fbn1^C1039G/+^) results in accelerated BBB degradation, increased inflammatory cytokines (TNF-α), MMPs −2/-9, and TGF-*β* in the MCA and choroid plexus (27). Overall, several preclinical studies utilize well-established animal models for EDS, MFS, and Loeys-Dietz Syndrome (LDS) to identify and optimize therapeutic approaches. These studies indicate specific signaling cascades in the development of EDS-related weakened vasculature, hypermobility, and cervical instability. The same signaling cascades are implicated in age-related weakened vasculature and cervical instability, leaving the elderly population more vulnerable to TBI, contributing to a hypothesis that patients with connective tissue disorders experience tissue-specific characteristics of accelerated aging (28, 29). Further, TBI can cause dysregulation of signaling cascades associated with dysfunction seen directly and indirectly in connective tissue disorders, leaving TBI patients more vulnerable to a second TBI. These similar signaling cascades represent potential targets for treatment in these various populations. It is noteworthy that current preclinical connective tissue disorder research in these models has primarily focused on peripheral manifestations associated with each disorder, with minimal neurophysiological investigation. This gap in research requires investigation to improve the quality of life for patients with connective tissue disorders, with potential impact beyond this patient population.

## Data Availability

The original contributions presented in the study are included in the article/Supplementary Material, further inquiries can be directed to the corresponding author/s.
